# Physical activity levels in cognitively normal and cognitively impaired oldest-old and the association with dementia risk factors: a pilot study

**DOI:** 10.1186/s12877-023-03814-4

**Published:** 2023-03-07

**Authors:** Marijn Muurling, Maryam Badissi, Casper de Boer, Nienke Legdeur, Frederik Barkhof, Bart N.M. van Berckel, Andrea B. Maier, Mirjam Pijnappels, Pieter Jelle Visser

**Affiliations:** 1 Department of Neurology, Amsterdam Neuroscience, Alzheimer Center Amsterdam, Vrije Universiteit Amsterdam, Amsterdam UMC, Amsterdam, The Netherlands; 2 Department of Radiology and Nuclear Medicine, Amsterdam Neuroscience, Vrije Universiteit Amsterdam, Amsterdam UMC, Amsterdam, The Netherlands; 3 Institutes of Neurology and Healthcare Engineering, University College London, London, UK; 4 Department of Medicine and Aged Care, @AgeMelbourne, The University of Melbourne, The Royal Melbourne Hospital, Parkville, 3050 VIC Australia; 5 Department of Human Movement Sciences, @AgeAmsterdam, Amsterdam Movement Science, Vrije Universiteit Amsterdam, Amsterdam, The Netherlands; 6 Healthy Longevity Program, Yong Loo Lin School of Medicine, National University of Singapore, Singapore, Singapore; 7 Centre for Healthy Longevity, @AgeSingapore, National University Health System, Singapore, Singapore; 8 Department of Psychiatry and Neuropsychology, School for Mental Health and Neuroscience, Maastricht University, Maastricht, The Netherlands; 9 Department of Neurobiology, Care Sciences and Society, Division of Neurogeriatrics, Karolinska Institutet, Stockholm, Sweden

**Keywords:** Cognitively impaired, 90+, Physically active, Physical performance, Brain pathology

## Abstract

**Background:**

Research assessing the relationship of physical activity and dementia is usually based on studies with individuals younger than 90 years of age. The primary aim of this study was to determine physical activity levels of cognitively normal and cognitively impaired adults older than 90 years of age (oldest-old). Our secondary aim was to assess if physical activity is associated with risk factors for dementia and brain pathology biomarkers.

**Methods:**

Physical activity was assessed in cognitively normal (N = 49) and cognitively impaired (N = 12) oldest-old by trunk accelerometry for a 7-day period. We tested physical performance parameters and nutritional status as dementia risk factors, and brain pathology biomarkers. Linear regression models were used to examine the associations, correcting for age, sex and years of education.

**Results:**

Cognitively normal oldest-old were on average active for a total duration of 45 (SD 27) minutes per day, while cognitively impaired oldest-old seemed less physically active with 33 (SD 21) minutes per day with a lower movement intensity. Higher active duration and lower sedentary duration were related to better nutritional status and better physical performance. Higher movement intensities were related to better nutritional status, better physical performance and less white matter hyperintensities. Longer maximum walking bout duration associated with more amyloid binding.

**Conclusion:**

We found that cognitively impaired oldest-old are active at a lower movement intensity than cognitively normal oldest-old individuals. In the oldest-old, physical activity is related to physical parameters, nutritional status, and moderately to brain pathology biomarkers.

**Supplementary Information:**

The online version contains supplementary material available at 10.1186/s12877-023-03814-4.

## Introduction

To reduce pressure on the health care systems with the ageing population, healthy ageing is becoming increasingly important [1]. Higher physical activity (PA) in older adults is associated with positive clinical outcomes [2] such as lower frailty [3], better muscle strength and power [4], lower Body Mass Index (BMI) [5, 6], and survival [7]. Moreover, an active lifestyle is associated with better global cognition [8] and lower risk for dementia, as shown in the World Health Organization (WHO) guidelines on risk reduction of cognitive decline and dementia [9, 10]. Even at low-intensity and when the PA guidelines are not met, PA is also associated with better preserved brain volumes [11] and less white matter hyperintensities (WMH) [12]. To reduce dementia risk, it is therefore recommended for older adults to stay physically active [9].

PA recommendations for dementia risk reduction are generally based on studies with individuals aged between 60 and 80 years of age. To date, little is known about the relationship between physical activity and dementia risk factors for the oldest-old, i.e., adults aged 90 years or older, while this special group is vulnerable for age-related physical impairments. Moreover, PA is usually measured using questionnaires such as the LASA Physical Activity Questionnaire [13], which may be unreliable: people tend to under- or overestimate their physical activity depending on for example their cognition [14], BMI, physical ability, and the presence of depressive symptoms [15]. Body-worn accelerometers enable objective measurement of physical activity in older and oldest-old adults and also provide insight in the type of activity (e.g. sitting, standing, walking or cycling), and the intensity of the activities [16]. Moreover, accelerometers are easy-to-use and relatively inexpensive, and, most importantly, are found to be acceptable and reliable for dementia patients [17]. In this research, we therefore used accelerometers to objectively measure physical activity in the oldest-old.

The primary aim of this cross-sectional study was to determine and compare physical activity levels of cognitively normal and cognitively impaired oldest-old individuals as assessed with body-worn accelerometers. We hypothesized that (mildly) cognitively impaired oldest-old are less physically active, both in duration and intensity, than cognitively normal oldest-old. Additionally, we assessed if cognitively normal oldest-old individuals who are physically more active, score lower on risk factors for dementia, e.g., physical parameters and nutritional status [18], and brain pathology biomarkers, e.g., white matter hyperintensities (WMH), hippocampal volume and amyloid binding [19]. These risk factors were chosen since they were found to be related to both cognitive impairment [18] and physical activity [2–6, 11, 12].

## Method

### Participants

**Fig. 1:**
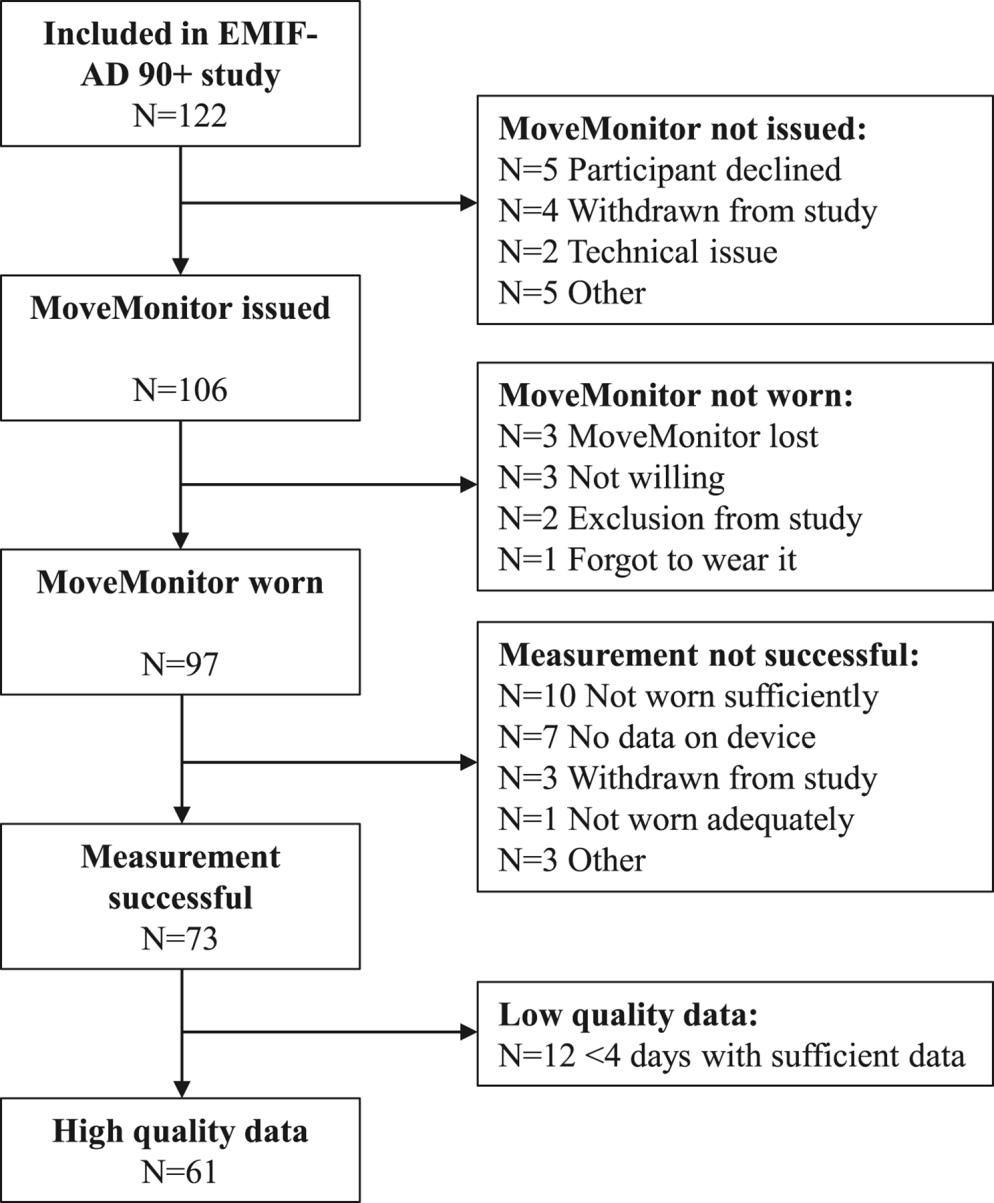
Flow-chart of the included participants in the current study from the EMIF-AD 90 + study

We included cognitively normal (CN, n = 49) and cognitively impaired (CI, n = 12) individuals from the European Medical Information Framework for AD (EMIF-AD) 90 + study [20]. Inclusion criteria for the cognitively normal group were older than 90 years of age, a global Clinical Dementia Rating (CDR) [21] score of 0, and a Mini-Mental State Examination (MMSE) [22] score ≥ 26. Inclusion criteria for the cognitively impaired group were older than 85 years of age, a global CDR score ≥ 0.5, a MMSE score of 20–28, and a clinical diagnosis of amnestic mild cognitive impairment (aMCI) [23] or probable or possible AD [24]. Exclusion criteria were severe depression, physical inability to undergo the procedures, visual or hearing impairment which made neuropsychological testing impossible and other comorbidities or medication that could impair cognition [20]. No additional in- and exclusion criteria were used for the current study as compared to the core EMIF-AD 90 + study. Since the use of the accelerometers was not a prerequisite to participate in the study, not all participants used the accelerometers. Figure 1 shows the reasons why only half of the EMIF-AD 90 + participants were included in the current study. All participants were recruited in Amsterdam UMC, The Netherlands.

### Study procedures

We collected data on demographics, cognition, functional abilities, physical performance, nutritional status and brain pathology biomarkers during two home visits and one or two hospital visits within three months [20]. Physical activity levels were assessed during a 7-day data collection period at the participant’s home.

### Clinical examination

The MMSE was registered during the first home visit. Functional impairment was assessed using the Amsterdam Instrumental Activities of Daily Living (IADL) questionnaire, which is an informant based scale aiming to detect IADL impairment in early dementia [25] with higher scores indicating less functional impairment. The questionnaire was sent to the informant by email. Missing values were caused by informants not filling in the questionnaire at home, despite repeated reminders. Comorbidities were registered using the Cumulative Illness Rating Scale – Geriatrics (CIRS-G) [26], with higher scores indicating more comorbidities.

### Physical activity

Physical activity was assessed using a triaxial accelerometer (DynaPort MoveMonitor, McRoberts B.V., The Hague, The Netherlands) with sampling frequency of 100 Hz. Participants were instructed to wear the MoveMonitor day and night for 7 days, except during showering and swimming, at the lower back by use of the supplied elastic belt around the waist. We only included participants who wore the MoveMonitor during the 7-day data collection period on average 75% of the day [27], of which at least four days were complete (worn > 94% of the day). Wearing time and activity classification (i.e., lying, sitting, standing, walking, cycling) were based on the manufacturer’s algorithms [28]. Based on these classifications, we calculated the following PA parameters for further analyses: total active duration (walking and cycling), total sedentary duration (lying and sitting), number of sitting bouts, the maximum walking bout duration and mean movement intensity (MI) of active periods. Number of steps was used to describe the physical activity level of the oldest-old, but was not included in the analyses, since number of steps was highly correlated with total active duration (r = 0.98). Each of these parameters have been shown to be substantially lower in the oldest-old [29]. For the descriptives, the duration times were not corrected for wearing days, since the wear-time was high in all participants (mean 163, SD 7, range 144–168 h per week) and to keep the numbers easy to interpret. However, the duration times were corrected for wearing time in the statistical analyses to keep the analyses as accurate as possible. Movement Intensity (MI) gives the mean body acceleration, calculated as the root sum square of the filtered triaxial accelerations for the corresponding activities [30], and gives an indication of the power of activities. The maximum walking bout duration is the longest walking period during the 7-day data collection period. Number of sitting bouts was negatively correlated to the mean duration of sitting bouts (r=-0.61), meaning that individuals that show higher number of sitting bouts sit less long, which is related to being more physically active.

### Physical parameters

Hand grip strength of the dominant hand was measured using a hand dynamometer (Jamar hand dynamometer; Sammons Preston, Inc., Bolingbrook, IL., USA) [31]. Each participant had two attempts to reach their maximal grip strength, and the highest attempt was used. Muscle mass index was calculated as the measured skeletal muscle mass in kilograms using a Bioelectrical Impedance Analysis (BIA; InBody 770; Biospace Co., Ltd., Seoul, Korea) divided by the participant’s squared height. The Short Physical Performance Battery (SPPB) is a battery to assess physical performance and included balance tests, a 4-meter walk and the chair stand test [32]. SPPB scores range from 0 to 12, with higher scores indicating better physical performance.

### Nutritional status

Nutritional status was assessed using BMI and the Mini-Nutritional Assessment (MNA). From the MNA, we used the assessment score only, which ranges from 0 to 16 with higher scores meaning better nutritional status [33].

### Brain pathology biomarkers

To obtain markers of neurodegeneration, participants underwent a magnetic resonance imaging (MRI) scan (CN: n = 42, CI: n = 10). 3D-T1 weighted, and 3D sagittal fluid-attenuated inversion recovery (FLAIR) images were acquired on a Philips 3T Achieva scanner, more details can be found in Pelkmans et al. [34]. Measures for WMH and hippocampal volume were retrieved from the MRI images as described in [18]. WMH and hippocampal volume were corrected for intracranial volume (%ICV).

To obtain amyloid status, participants underwent a dynamic [^18^F] flutemetamol amyloid-PET scan (CN: n = 40, CI: n = 9). The scans were performed on a Philips Ingenuity TF PET-MRI scanner (Philips Medical Systems, Cleveland, OH, USA). Parametric nondisplaceable binding potential (BP_ND_) images were generated, see Pelkmans et al. [34] for more details. Images were visually assessed by three independent and well-trained readers, who were blinded to the clinical and demographic data, as either amyloid negative (Aβ-) or amyloid positive (Aβ+). For the regression models, global BP_ND_ was used [18].

### Statistical analysis

Statistical analyses were performed in R (version 4.0.3). We compared demographics between the cognitively normal and cognitively impaired groups using independent t-tests or Wilcoxon signed-rank tests when appropriate. Physical activity parameters were compared between the cognitively normal and cognitively impaired groups using linear models, corrected for age, sex and years of education. To assess if physical activity is related to dementia risk factors in cognitively normal oldest-old, we used 40 regression models, with as independent variable one physical activity variable (either total active duration, total sedentary duration, total sitting bouts, maximum walking bout duration, and mean movement intensity) and as dependent variable one dementia risk factor (either handgrip strength, muscle mass index, SPPB, MNA, or BMI) or brain pathology biomarker (either WMH, hippocampal volume or amyloid binding), corrected for age, sex and years of education. Only data of cognitively normal participants were included in these analyses. For the models including WMH and hippocampal volume, the analysis included participants with a valid MRI scan, while for the models including amyloid binding, the analysis included participants with a valid PET scan (Table 1). All variables were z-transformed to compare the regression models. The physical activity parameters total active duration, total sedentary duration and total sitting bouts were normalized to the total wearing time. The variables total wear time, total active duration, maximum walking bout duration, grip strength, WMH and amyloid binding were log transformed since the data was skewed. To correct for multiple testing, the p-values from the regression analyses were corrected using False Discovery Rate (FDR) correction [35] per outcome. As exploratory analyses, the amyloid positive and amyloid negative groups, in cognitively normal oldest-old only, were compared using linear models, corrected for age, sex and years of education. A p-value < 0.05 was considered significant.

**Table 1:** Characteristics of the cognitively normal and cognitively impaired groups

	Total		Cognitively normal		Cognitively impaired		P-value between groups
	N	M (SD) / n (%)	N	M (SD) / n(%)	N	M (SD) / n (%)	
**Clinical characteristics**							
Age, y	61	92.3 (2.0)	49	92.5 (1.8)	12	91.7 (2.6)	p = 0.24
Female, n(%)	61	30 (49%)	49	22 (45%)	12	8 (67%)	p = 0.30
Education, y	61	13.3 (5.1)	49	13.3 (5.0)	12	13.2 (5.6)	p = 0.86
Lives independently, n(%)	61	56 (92%)	49	46 (94%)	12	10 (83%)	p = 0.54
Amyloid positive, n(%)	49	22 (45%)	40	16 (40%)	9	6 (67%)	p = 0.28
MMSE	61	27.7 (2.6)	49	28.6 (1.3)	12	23.9 (3.1)	p < 0.001***
Amsterdam IADL	38	57.4 (10.7)	31	60.5 (7.3)	7	43.4 (12.7)	p = 0.002**
Comorbidity, CIRS-G	60	8.6 (3.6)	48	8.3 (3.6)	12	9.4 (3.6)	p = 0.36
**Physical activity**							
Total worn, h	61	162.5 (6.6)	49	162.1 (6.7)	12	164.2 (6.2)	P = 0.49
Total active, h	61	5.0 (3.0)	49	5.3 (3.1)	12	3.9 (2.5)	p = 0.12
Total sedentary, h	61	141.1 (9.1)	49	140.7 (9.4)	12	142.7 (7.8)	p = 0.40
Number of sitting bouts	61	729.4 (335.9)	49	741.1 (355.4)	12	681.5 (247.1)	p = 0.30
Maximum walking bout duration, min	61	6.3 (7.2)	49	6.7 (7.9)	12	4.5 (2.9)	p = 0.40
MI moving, mg	61	147.3 (26.2)	49	149.6 (26.5)	12	137.9 (24.0)	p = 0.03*
**Physical parameters**							
Handgrip strength, kg	60	16.9 (7.6)	48	18.2 (7.8)	12	11.4 (4.0)	p = 0.004**
Muscle mass index, kg/m^2^	56	9.2 (1.1)	45	9.3 (1.0)	11	8.6 (1.1)	p = 0.09
SPPB, points	60	8.0 (2.6)	48	8.3 (2.7)	12	6.7 (1.8)	p = 0.04*
**Nutritional status**							
MNA, points	60	12.8 (1.5)	49	12.9 (1.5)	11	12.3 (1.3)	p = 0.15
BMI, kg/m^2^	61	25.7 (3.5)	49	25.7 (3.6)	12	25.6 (3.2)	p = 0.92
**Brain pathology biomarkers**							
WMH volume, %ICV	51	1.35 (0.96)	41	1.26 (0.91)	10	1.68 (1.14)	p = 0.26
Hippocampal volume, %ICV	52	0.19 (0.03)	42	0.20 (0.03)	10	0.17 (0.04)	p = 0.06
Amyloid load	44	0.33 (0.29)	35	0.31 (0.30)	9	0.41 (0.21)	P = 0.18

Note. The N-columns display the number of participants for which the data is available. Total duration variables are given as the total duration during the 7-day data collection. MI moving gives the average body acceleration over all moving periods during the 7-day data collection. Lives independently means that that participant lives not in a nursing home or hospital. Group comparisons from the demographic variables are tested with t-tests or Wilcoxon signed-rank tests, while the physical activity variables are tested with regression models adjusted for age and sex. Abbreviations: BMI, Body Mass Index; CIRS-G, Cumulative Illness Rating Scale – Geriatrics; ICV, intracranial volume; MI, Movement Intensity; MMSE, Mini-Mental State Examination; MNA, Mini-Nutritional Assessment; SPPB, Short Physical Performance Battery; WMH, White Matter Hyperintensities; kg, kilograms; m, meter; h, hour; mg, milli-body acceleration (m/s^2^ *10^− 3^); y, years. *p < 0.05, **p < 0.01, ***p < 0.001.

**Table 2:** Associations between physical activity measures and risk factors for dementia in cognitively normal oldest-old individuals

	Total active duration	Total sedentary duration	Number of sitting bouts	Maximum walking bout duration	MI moving
Grip strength	0.13 (0.11), p = 0.27	-0.13 (0.10), p = 0.24	0.27 (0.09), p = 0.02*	0.10 (0.10), p = 0.45	0.22 (0.11), p = 0.08
Muscle mass	-0.16 (0.12), p = 0.27	0.24 (0.11), p = 0.05	-0.14 (0.11), p = 0.24	-0.03 (0.12), p = 0.84	0.09 (0.13), p = 0.54
SPPB	0.34 (0.13), p = 0.057	-0.20 (0.13), p = 0.16	0.24 (0.12), p = 0.13	0.35 (0.12), p = 0.04*	0.56 (0.12), p < 0.001***
MNA	0.33 (0.15), p = 0.08	-0.41 (0.14), p = 0.02*	0.30 (0.14), p = 0.10	0.39 (0.14), p = 0.04*	0.41 (0.15), p = 0.04*
BMI	-0.46 (0.16), p = 0.04*	0.51 (0.14), p = 0.006**	-0.46 (0.14), p = 0.02*	-0.25 (0.16), p = 0.23	-0.17 (0.17), p = 0.44
WMH	-0.16 (0.19), p = 0.40	0.11 (0.18), p = 0.54	-0.20 (0.17), p = 0.26	-0.24 (0.19), p = 0.32	-0.43 (0.17), p = 0.04*
Hippocampal volume	0.20 (0.15), p = 0.27	-0.26 (0.14), p = 0.12	0.18 (0.14), p = 0.24	0.10 (0.16), p = 0.61	0.18 (0.15), p = 0.38
Amyloid binding	0.39 (0.17), p = 0.08	-0.41 (0.17), p = 0.05	0.25 (0.18), p = 0.24	0.45 (0.18), p = 0.049*	0.12 (0.21), p = 0.56

Note. Outcomes are given as β(SE). P-values are FDR corrected. All variables are z-transformed, so that betas are comparable between variables. All analyses are corrected for age, sex and years of education. Abbreviations: BMI, Body Mass Index; MNA, Mini-Nutritional Assessment; SPPB, Short Physical Performance Battery; MI, Movement Intensity; WMH, White Matter Hyperintensities. *p < 0.05, **p < 0.01, ***p < 0.001.

## Results

### Demographics

The cognitively normal and cognitively impaired groups did not differ in age, sex distribution, years of education and level of comorbidities (Table 1). Over 80% in both groups lived independently, while approximately 50% in both groups received home care, e.g., help with cleaning, showering or preparing dinner. In the cognitively impaired group, 67% (n = 6) were amyloid positive compared to 40% (n = 16) in the cognitively normal group. In the cognitively impaired group, 4 participants had a clinical diagnosis MCI and 8 participants a clinical diagnosis of dementia.

### Physical activity levels in the oldest-old

Compliance of wearing the MoveMonitor was high: participants wore the MoveMonitor on average 163 (SD 6.7) hours during the 168-hour data collection period. The cognitively normal oldest-old were on average active for a total duration of 45 (SD 27, range 1-114) minutes per day (Table 1) with on average 3395 (SD 2259, range 24-9123) steps per day. Maximum walking bout duration was 6.7 (SD 7.9, range 0.3–45.7) minutes. On average, participants had 106 (SD 51, range 20–287) sittings bouts per day.

**Fig. 2:**
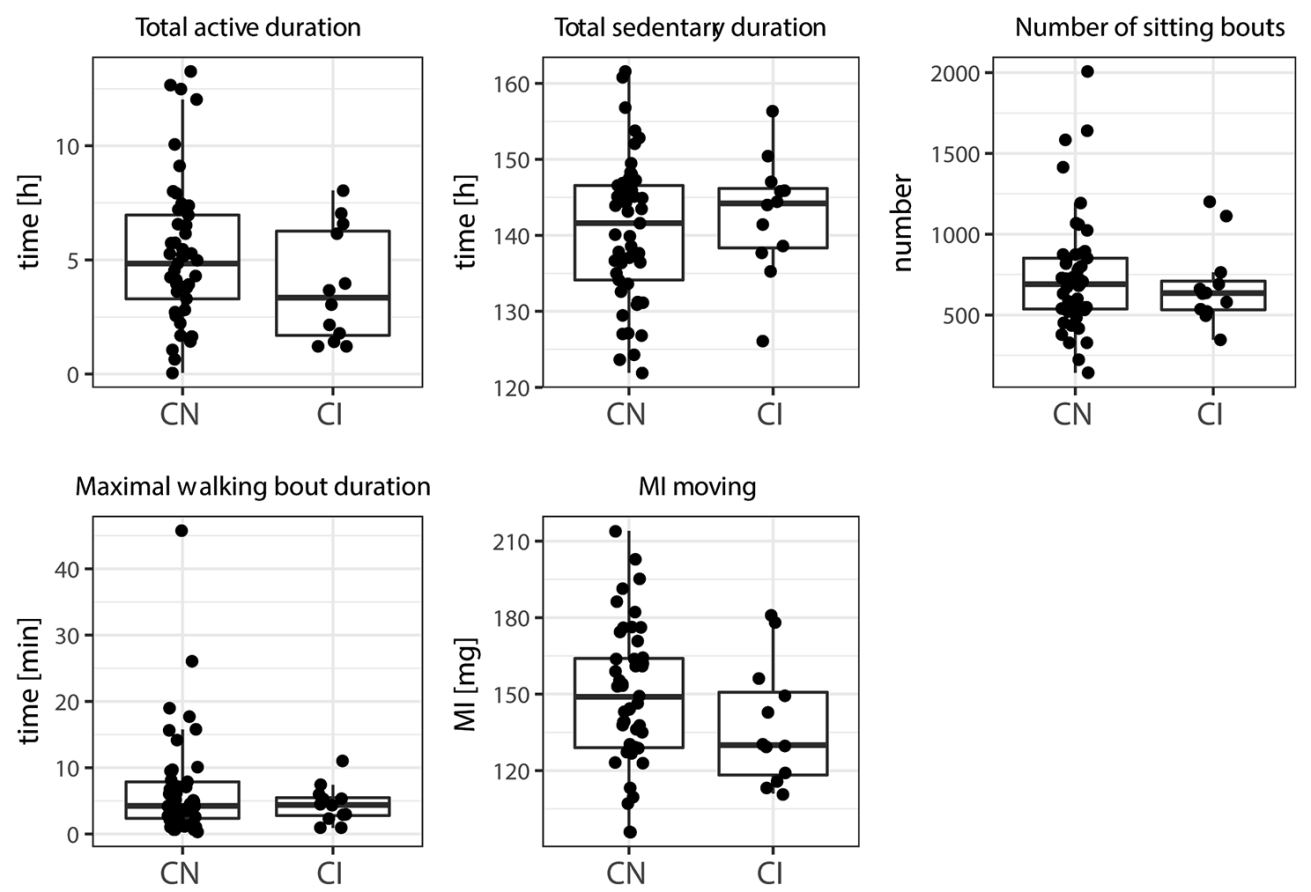
Comparisons between the cognitively normal (CN) and cognitively impaired (CI) groups on 5 physical activity variables. Total duration variables are given as the total duration during the 7-day data collection in hours. MI moving gives the average body acceleration over all moving periods during the 7-day data collection. Each dot represents the data of one participant. Abbreviations: CN, cognitively normal; CI, cognitively impaired; MI, Movement Intensity; h, hour; mg, milli-body acceleration (m/s^2^ *10^− 3^)

Compared to cognitively normal oldest-old, cognitively impaired participants had lower movement intensities during active periods (Table 1; Fig. 2). Total active duration, total sedentary duration, number of sitting bouts and maximum walking bout duration did not differ between cognitively normal and cognitively impaired participants.

### Association of physical activity with dementia risk factors and pathology in cognitively normal individuals

**Fig. 3:**
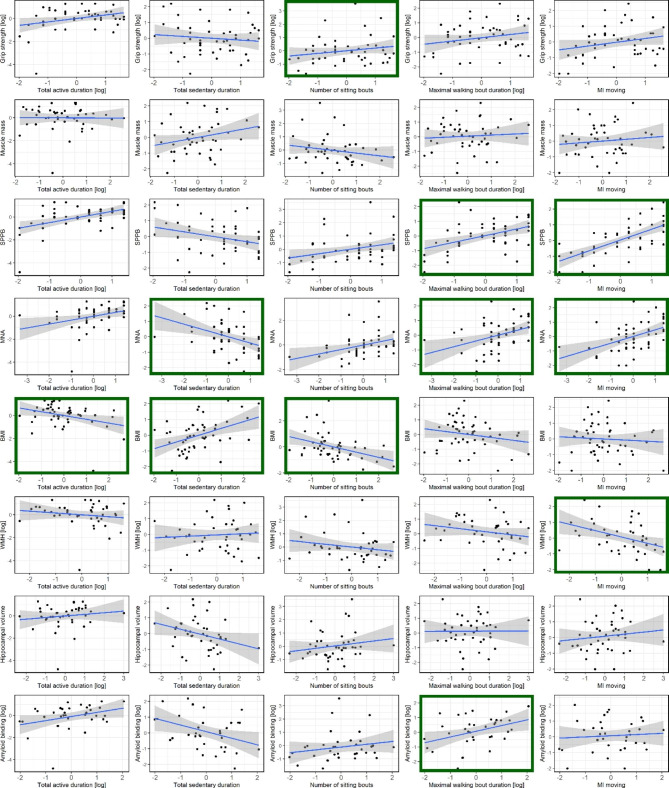
Scatterplots and regression lines for all tested regressions as shown in Table 2, between the z-scored physical activity measures and z-scored dementia risk factors or brain pathology biomarkers in cognitively normal older adults. Regression lines are uncorrected for age, sex and years of education. Green squares indicate the significant relations after correction for multiple testing from Table 2. Abbreviations: BMI, Body Mass Index; MNA, Mini-Nutritional Assessment; SPPB, Short Physical Performance Battery; MI, Movement Intensity; WMH, White Matter Hyperintensities

In cognitively normal oldest-old individuals, higher total active duration was associated with lower BMI (Table 2; Fig. 3). Lower duration of total sedentary time associated with higher MNA scores and lower BMI. Higher number of sitting bouts associated with lower BMI and higher handgrip strength. Higher maximal walking bout duration associated with higher SPPB scores, higher MNA scores and higher amyloid binding. Higher movement intensities were related to higher SPPB scores, higher MNA scores and less WMH.

Since amyloid binding was found to be related to maximal walking bout duration, the amyloid positive and amyloid negative groups were compared as exploratory analyses. The amyloid negative group was higher educated and had a higher BMI (Supplementary Table 1). The amyloid negative group also showed higher sedentary duration. No other differences were found.

## Discussion

The aim of this study was to determine physical activity levels of cognitively normal and cognitively impaired oldest-old, using body-worn accelerometers, and to associate physical activity with dementia risk factors and brain pathology biomarkers in cognitively normal oldest-old. We found that cognitively impaired oldest-old are active at a lower movement intensity than cognitively normal oldest-old. Being more physically active and showing less sedentary behavior was associated with better nutritional status, and higher movement intensities were associated with better physical performance, better nutritional status, and lower white matter hyperintensities.

### Physical activity in cognitively normal oldest-old participants

Physical activity levels of cognitively normal oldest-old are relatively low: on average, cognitively normal oldest-old are physically active 45 min per day and are inactive (i.e., sitting or lying) the rest of the day. The physical activity levels in the current study are similar to the results of the oldest age group found by a large study investigating the physical activity levels of different age groups [29]. Even though their oldest age group was somewhat younger (mean age 85 years) than our sample, both studies found similar average movement intensity, active duration, and maximum walking bout duration.

The current physical activity recommendations for older adults by the WHO are at least 150 to 300 min of moderate-intensity aerobic physical activity or 75–150 min vigorous-intensity aerobic physical activity, and muscle-strengthening activities on two days per week [36]. In our study, 16% of the cognitively normal participants were less than 150 min per week physically active, which was less than the world-wide average of 31% of people not meeting the recommendations [37]. However, movement intensity during these active bouts was relatively low: the average movement intensity for the cognitively normal group (0.147 m/s^2^) is associated with a walking speed of 2–3 km/h, according to a validation test with the MoveMonitor [30]. Moreover, the total number of steps was low as well: a recommended number of steps/day is 10,000 – or at least 7000 step/day for healthy adults older than 65 years [38] – while our cognitively normal participants showed on average 3395 steps/day. Although step count may be underestimated by the MoveMonitor [30, 39], particularly in shuffling gait as common in the oldest old, and the number of steps is much higher than the 402 steps/day for frail geriatric patients [40], we can state that the majority of our sample did not meet the WHO physical activity recommendations. It is expected that physical activity is even lower for the entire oldest-old population, since one of the inclusion criteria for this study was that participants were able to visit the clinic, which could have led to a selection bias of physically stronger oldest-old.

### Physical activity in cognitively impaired compared to cognitively normal oldest-old individuals

Compared to cognitively normal oldest-old, cognitively impaired oldest-old were active with a lower movement intensity, which is in line with previous research [41]. From our data, it cannot be concluded whether low activity levels are cause or effect of cognitive decline, i.e., do people start to be less physically active because of their cognitive decline or does low physical activity lead to more or faster cognitive decline? Previous prospective studies have shown that physical activity decreases the risk for cognitive decline [8, 42]. Still, because neurodegenerative disorders have a long preclinical stage, in which cognitive impairments are not present, it cannot be excluded that the decreased physical is a result of the early neurodegeneration, in particular if the follow-up is shorter than 10 years [43]. Therefore, further longitudinal research is needed that assesses physical activity and risk of dementia with a long follow-up time, e.g. over 20 years.

### Association of physical activity with dementia risk factors

Lower total sedentary duration and more – and thus shorter – sitting bouts were associated with better nutritional status and better grip strength, while moving with a higher intensity was associated with better physical performance, nutritional status and lower white matter hyperintensities. These findings have already been shown in a younger population [44], and it is a major finding that these associations are present in the oldest-old population as well. Our results provide evidence that people should be motivated to stay physically active, even at an old age. However, it is unknown how physically active the participants were during early life, and the results found in this study are potentially the result of participants being physically active at a younger age and staying active during their life [45].

Physical activity levels were only moderately related to brain pathology biomarkers. Higher levels of movement intensities were associated to less white matter hyperintensities. These findings are in accordance with multiple studies with a younger population reviewed by [12], and can be explained by the fact that physical activity improves vascular risk factors. Longer maximal walking bouts were associated with more amyloid binding, which was unexpected. Therefore, as exploratory analyses, cognitively normal amyloid positive oldest-old were compared with cognitively normal amyloid negative oldest-old, using the same methods as the comparison between cognitively normal and cognitively impaired oldest-old. Unexpectedly, amyloid negative oldest-old showed more sedentary behavior, but this can be explained by the higher BMI of this group. The results need to be interpreted with caution because of the small sample size (N = 35 for amyloid binding and N = 41 for WMH).

### Strengths and Limitations

This study is unique in the field because of the old population and the objective and detailed measurement of physical activity with inertial sensors during daily routines, but it has its limitations as well. Firstly, the sample size was relatively small. Future studies with larger sample sizes replicating our results would therefore be helpful. More specifically, the number of cognitively impaired participants was low. Upon inclusion, the group was larger, but dementia participants had trouble wearing the MoveMonitor, resulting in only 12 participants with enough valid recording days, which could have led to a selection bias and overestimation of the physical activity levels of cognitively impaired oldest-old. However, the wear time in this group was similar to the wear time of the cognitively normal group. Secondly, our results are cross-sectional, which means that we cannot address causality. Finally, our cognitively impaired group is heterogeneous; both MCI and dementia patients are included, and for several participants the etiology was unknown. Moreover, the majority of participants experiences co-morbidities which could have affected physical outcome. This heterogeneity is, however, characteristic for this oldest-old population and is therefore not likely to affect the generalizability of the study.

## Conclusion

In this study, we showed that cognitively normal oldest-old individuals do not meet age-adjusted physical activity recommendations and that cognitively impaired oldest-old are even less physically active than cognitively normal oldest-old individuals. We confirmed previous research on the relation of physical activity levels with dementia risk factors and white matter hyperintensities, but now in an older population. Further research should use larger sample sizes and longitudinal analyses to assess if high physical activity can lead to slower cognitive and functional decline.

## Electronic supplementary material

Below is the link to the electronic supplementary material.

Supplementary Material 1. Supplementary Table 1: Characteristics of the amyloid positive and amyloid negative groups.

## Data Availability

The datasets used and/or analyzed during the current study are available from the corresponding author on reasonable request.
