# Supplementary material for: Physical activity levels in cognitively normal and cognitively impaired oldest-old and the association with dementia risk factors: a pilot study
Source: BMC Geriatr. 2023 Mar 7;23:129. doi: 10.1186/s12877-023-03814-4 (PMC9993554; doi:10.1186/s12877-023-03814-4)
Supplement: Supplementary file 1 — Supplementary Material 1. Supplementary Table 1: Characteristics of the amyloid positive and amyloid negative groups. [file 12877_2023_3814_MOESM1_ESM.docx]

Supplementary Table 1: Characteristics of the amyloid positive and amyloid negative groups

|  | **Total** | | **Amyloid positive** | | **Amyloid negative** | | **P-value between groups** |
| --- | --- | --- | --- | --- | --- | --- | --- |
|  | N | M(SD) / n(%) | N | M(SD) / n(%) | N | M(SD) / n(%) |  |
| **Clinical characteristics** | | | | | | | |
| Age, y | 35 | 92.4 (1.9) | 14 | 92.6 (2.0) | 21 | 92.3 (1.9) | p=0.57 |
| Female, n(%) | 35 | 18.0 (51%) | 14 | 9 (64%) | 21 | 9 (43%) | p=0.37 |
| Education, y | 35 | 13.0 (4.6) | 14 | 11.2 (4.5) | 21 | 14.1 (4.5) | p=0.04* |
| Lives independently, n(%) | 35 | 32 (91%) | 14 | 12 (86%) | 21 | 20 (95%) | p=0.71 |
| Amyloid positive, n(%) | 35 | 14 (40%) | 14 | 14 (100%) | 21 | 0 (0%) | p<0.001*** |
| MMSE | 35 | 28.6 (1.4) | 14 | 28.0 (1.2) | 21 | 29.0 (1.3) | p=0.01* |
| Amsterdam IADL | 22 | 59.2 (7.9) | 7 | 55.7 (9.2) | 15 | 60.8 (6.9) | p=0.18 |
| Comorbidity, CIRS-G | 34 | 7.9 (3.2) | 14 | 7.6 (4.0) | 20 | 8.0 (2.6) | p=0.36 |
| **Physical activity** | | | | | | | |
| Total worn, h | 35 | 162.9 (6) | 14 | 161.6 (7.3) | 21 | 163.7 (4.9) | p=0.18 |
| Total active, h | 35 | 5.2 (3.4) | 14 | 6.1 (3.8) | 21 | 4.6 (3.0) | p=0.09 |
| Total sedentary, h | 35 | 142.0 (9.0) | 14 | 137.6 (6.4) | 21 | 144.9 (9.4) | p=0.01* |
| Number of sitting bouts | 35 | 705.5 (387.9) | 14 | 700.1 (297.9) | 21 | 709.0 (445.1) | p=0.68 |
| Maximum walking bout duration, min | 35 | 6.8 (6.1) | 14 | 8.2 (6.7) | 21 | 5.8 (5.6) | p=0.08 |
| MI moving, mg | 35 | 151.3 (26.0) | 14 | 146.9 (23.0) | 21 | 154.2 (27.9) | p=0.79 |
| **Physical parameters** | | | | | | | |
| Handgrip strength, kg | 34 | 17.1 (8.0) | 13 | 14.3 (7.0) | 21 | 18.9 (8.3) | p=0.11 |
| Muscle mass index, kg/m^2^ | 34 | 9.2 (1.1) | 14 | 8.8 (1.1) | 20 | 9.5 (1.0) | p=0.05 |
| SPPB, points | 35 | 8.2 (2.9) | 14 | 7.9 (2.8) | 21 | 8.4 (2.9) | p=0.59 |
| **Nutritional status** | | | | | | | |
| MNA, points | 35 | 12.9 (1.6) | 14 | 12.8 (1.9) | 21 | 12.9 (1.4) | p=0.96 |
| BMI, kg/m^2^ | 35 | 25.9 (3.6) | 14 | 24.2 (2.3) | 21 | 27 (3.9) | p=0.01* |
| **Brain pathology biomarkers** | | | | | | | |
| WMH volume, %ICV | 34 | 1.32 (0.93) | 14 | 1.68 (1.2) | 20 | 1.07 (0.61) | p=0.22 |
| Hippocampal volume, %ICV | 34 | 0.2 (0.03) | 14 | 0.2 (0.04) | 20 | 0.2 (0.03) | p=0.85 |
| Amyloid load | 35 | 0.3 (0.3) | 14 | 0.6 (0.3) | 21 | 0.1 (0.1) | p<0.001*** |

Note. The N-columns display the number of participants for which the data is available. Total duration variables are given as the total duration during the 7-day data collection. MI moving gives the average body acceleration over all moving periods during the 7-day data collection. Lives independently means that that participant lives not in a nursing home or hospital. Group comparisons from the demographic variables are tested with t-tests or Wilcoxon signed-rank tests, while the physical activity variables are tested with regression models adjusted for age and sex. Abbreviations: BMI, Body Mass Index; CIRS-G, Cumulative Illness Rating Scale – Geriatrics; ICV, intracranial volume; MI, movement intensity; MMSE, Mini-Mental State Examination; MNA, Mini-Nutritional Assessment; SPPB, Short Physical Performance Battery; WMH, White Matter Hyperintensities; kg, kilograms; m, meter; h, hour; mg, milli-body acceleration (m/s^2^ *10^-3^); y, years. *p<.05, **p<.01, ***p<.001.
